# The Density of Cell Nuclei at the Materno-Fetal Exchange Barrier is Sexually Dimorphic in Normal Placentas, but not in IUGR

**DOI:** 10.1038/s41598-019-38739-9

**Published:** 2019-02-20

**Authors:** Nirav Barapatre, Eva Haeussner, David Grynspan, Christoph Schmitz, Franz Edler von Koch, Hans-Georg Frank

**Affiliations:** 1 LMU Munich, Faculty of Medicine, Institute of Anatomy, Chair of Neuroanatomy, Munich, Germany; 2 University of Ottawa, Department of Pathology and Laboratory Medicine, Ottawa, Canada; 3 Clinic for Obstetrics and Gynecology Dritter Orden, Munich, Germany

## Abstract

Placental sexual dimorphism is of special interest in prenatal programming. Various postnatal diseases with gender dependent incidence, especially neuropsychiatric disorders like schizophrenia and autism spectrum disorders, have prenatal risk factors established. However, the functional relevance of placental microarchitecture in prenatal programming is poorly investigated, mainly due to a lack of statistically efficient methods. We hypothesized that the recently established 3D microscopic analysis of villous trees would be able to identify microscopic structural correlates of human placental sexual dimorphism. We analyzed the density of cell nuclei of villous trophoblast, i.e. the materno-fetal exchange barrier, in placentas from term pregnancies. The cell nuclei were grouped into proliferative and non-proliferative nuclei by detection of a proliferation marker (PCNA). Normal female placentas showed a higher density of non-proliferating nuclei (PCNA-negative) in villous trophoblast than normal male placentas. The density of PCNA-negative cell nuclei was higher in placentas of pregnancies with intrauterine growth retardation (IUGR) than in control placentas. The data of the present study shows that the density of non-proliferative cell nuclei in the syncytial layer of villous trophoblast is influenced by fetal sex and by IUGR, while proliferation remains unchanged. A novel concept of post-fusion regulation of syncytial structure and function is proposed.

## Introduction

It is a well-established fact that the prenatal environment programs vulnerability for severe diseases in later life. Originally, this was discovered for ischemic heart disease in English residents^1,2^. This finding could be confirmed in many other countries^3–6^ and was extended to other diseases by studies on special populations including e.g. the Dutch Hunger Winter of 1944^7^, Swedish population cohorts^8^ and the Helsinki Birth Cohort^9^. Conditions influenced by prenatal factors include arterial hypertension^2,10^, type 2 diabetes^11^, obesity^12^, osteoporosis^13^, and neurological function^14,15^ including neuropsychiatric diseases like schizophrenia and autism spectrum disorder^16,17^. Many of these conditions, especially the neuropsychiatric syndromes, have a sex-biased incidence in postnatal life. The impact of placental sexual dimorphism on these postnatal health trajectories is an actual focus of research^16^.

Indeed, female and male placentas are genetically and phenotypically different^16,17^. The mean placental weight of term placentas is on average about 2% higher in normal male placentas than in normal female placentas^18^. Boys grow faster during pregnancy than girls and stimulate their mothers to a higher daily calorie intake^19^. The more accelerated growth of boys in the womb puts them at a higher risk for fetal demise compared to girls in case of adversity^20^. This is also confirmed by a publication on the Chinese Great Leap Forward famine^21^, reporting a higher intrauterine death rate of boys under maternal famine stress. Intrauterine growth retardation (IUGR) has a higher incidence in pregnancies with male than with female fetuses^22,23^. That pregnancies with male fetuses are at higher risk of adverse outcome than those with female fetuses underscores the clinical relevance of placental sexual dimorphism, and its impact on prenatal programming^16,24,25^.

During gestation, the biochemical indicators in maternal serum that show fetal/placental sex-dependent variation in concentrations include human chorionic gonadotropin (hCG)^26^, soluble fms-like tyrosine kinase-1 (sFlt-1)^27^ and placental alkaline phosphatase (PLAP)^28^. These placental proteins and angiogenic factors show elevated levels in maternal serum of healthy female pregnancies compared to male pregnancies.

The placental proteins hCG and PLAP as well as angiogenic factors like sFlt-1 are products of the trophoblast^29,30^. In light of these findings and recent data on thickness variability of the materno-fetal exchange barrier after prenatal stress^31^, the villous trophoblast qualifies as a candidate source tissue of placental structural and functional sexual dimorphism. Villous trophoblast is a bi-layered epithelial tissue with a biologically unique apical syncytial layer^30,32^, the workhorse of materno-fetal transfer processes.

Taken together, though there is substantial evidence of sexual dimorphism in human placentation with significant impact on life-long health, no morphologically recognizable sexually dimorphic tissue could be identified thus far at the microscopic level in placentas at birth. From gene expression studies it is known that the tissues of the placentas do have tissue specific sexually different gene expression patterns (“sexomes”)^33^. Spotting the site of sexual dimorphism is thus essential to reliably connect the genetic^16^, biochemical (placental proteins)^26–28^ and gross morphological (placental weight and birthweight)^17,18^ sex-specific differences of human placentas to a functional picture by anchoring them to a structure/tissue.

Microscopic investigation of the human placenta is not routinely being used in studies of prenatal programming or to analyze placental sexual dimorphism. This may be due to methodological shortcomings of contemporary, two-dimensional (2D) histologic analysis of placental sections^34^. Specifically, 2D histology of the human placenta inevitably leads to a loss of orientation in the three-dimensional (3D) structure of the human placenta. The placental functional microarchitecture consists of tree-like 3D structures called villous trees. These trees have fetal vessels and stroma internally and carry on their surface the villous trophoblast, an epithelial tissue of early embryonic origin that defines the materno-fetal exchange barrier^30^. Maturation and adaptation of branching of these trees occurs in 3D, an aspect that is essential for their biological interpretation. The loss of topologic orientation in 3D which is associated with 2D histology is especially relevant for tree structures which can only be fully interpreted in 3D. This shortcoming was overcome by the introduction of next-generation quantitative 3D histology of the human placenta, which guarantees instantaneous orientation in 3D and connects any identifiable structure like trophoblast cell nuclei to their 3D site of occurrence in the villous tree^35,36^.

In the present study we used this novel 3D microscopic approach^35,36^ to analyze villous trees of clinically normal pregnancies (normal) and placentas of pregnancies with intrauterine growth retardation (IUGR) at a challenging but manageable workload. We focused our study on all nuclei of villous trophoblast (i. e. cyto- and syncytiotrophoblast) occurring in terminal (bT0) and preterminal (bT1) peripheral branches^35,36^, which are a technically feasible target for 3D microscopy. Since differentiation of cyto- and syncytiotrophoblastic nuclei is a genuine 2D-microscopic feature, we used the proliferation marker PCNA as an observer-independent proxy to this. Most of the PCNA-positive nuclei will be cytotrophoblast nuclei, while most of the PCNA-negative nuclei will be syncytial^35^. For the first time we show, that the density of PCNA-negative (i.e. post-proliferative) cell nuclei of villous trophoblast is a physiologically sexually dimorphic feature situated directly at the functional heart of the human placenta, its materno-fetal exchange barrier.

## Results

**Figure 1:**
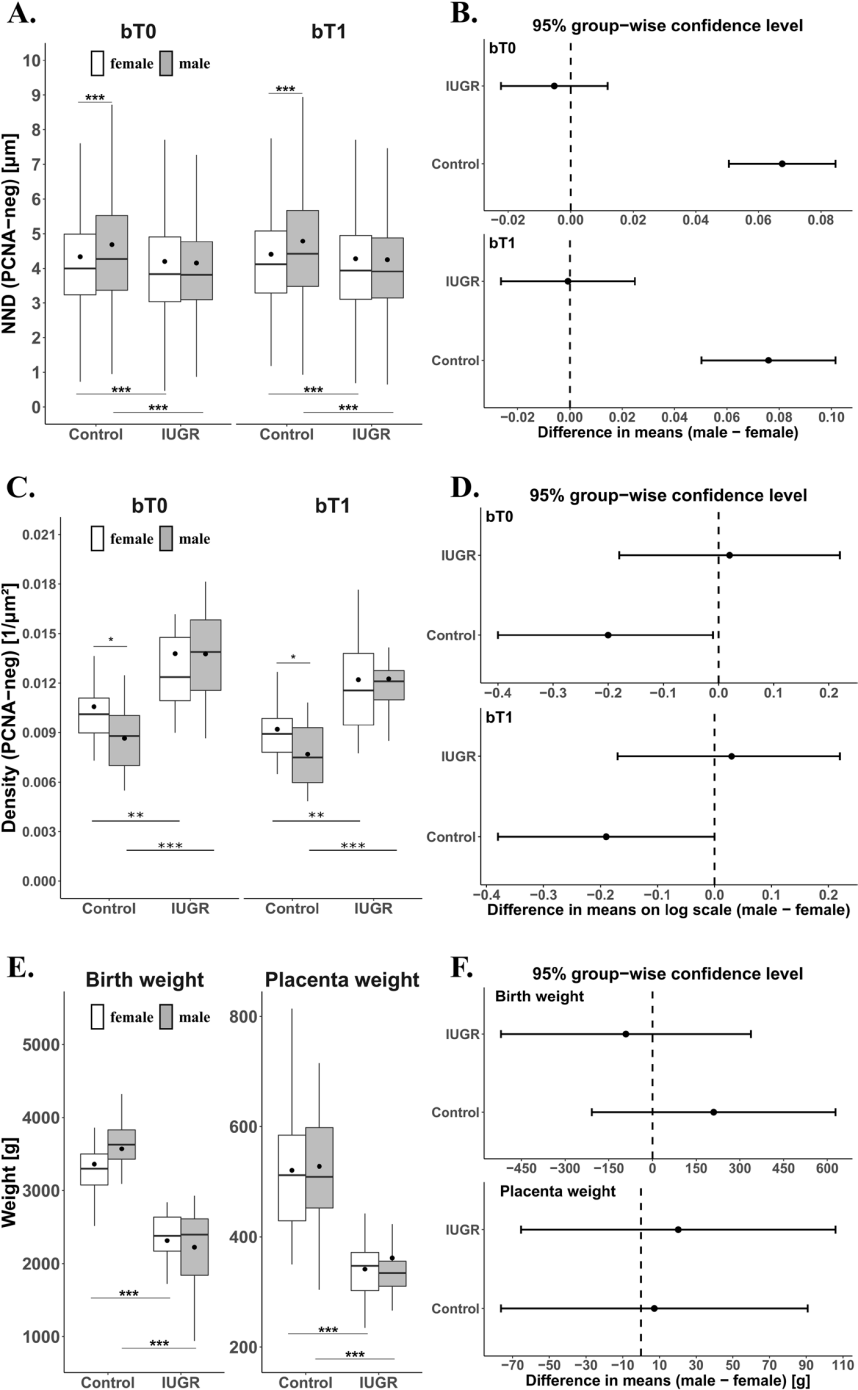
The density of cell nuclei of villous trophoblasts is graphed as Nearest Neighbor Distance (NND, **A**,**B**) and Mean Density (MD, **C**,**D**). The gross morphological parameters birth weight and placenta weight are shown in (**E**,**F**). The data are depicted by means of Tukey plots (**A**,**C**,**E**) and confidence intervals (**B**,**D**,**F**). The Tukey plots are grouped by clinical diagnosis groups (Control, IUGR), where Control represents clinically normal placentas. Males are represented by grey boxes. The sample mean is represented by a solid dot within the box. Levels of statistical significance are indicated by asterisks (*p < 0.05, **p < 0.01, ***p < 0.001) above the Tukey plots for sexual dimorphism and below the Tukey plots for comparison of clinical groups. (**A**) The figure depicts the NND of PCNA-negative (PCNA-neg) cell nuclei of villous trophoblast of terminal (bT0) and preterminal (bT1) branches of the villous tree (**B**). Confidence intervals (95%) are shown for the difference (male-female) in means of the logarithmic data (**C**). The figure depicts MD (MD as number of cell nuclei by surface area and branch) of cell nuclei of villous trophoblast of terminal (bT0) and preterminal (bT1) branches of the villous trees (**D**). Confidence intervals (95%) are shown for the difference (male-female) in means. (**E**) The figure depicts the birth weights and placenta weights of the present study (**F**). Confidence intervals (95%) are shown for the difference (male-female) in means.

42 placentas (23 female and 19 male) from clinically normal pregnancies were analyzed. The mean age of mothers was 33.9 ± 4.0 years. The mean gestational age was 39.3 ± 1.0 weeks. 40 placentas (22 female and 18 male) from pregnancies with intrauterine growth restriction (IUGR) were analyzed. The mean age of mothers was 35.5 ± 5.5 years. The mean gestational age was 37.5 ± 2.7 weeks. Apart from gestational age and birth weight, Table 1 lists the mean values for various other gross placental morphological parameters. With a sample size of 82 placentas in the present study, no gender specific differences were observed in birth weight or placental weight (Fig. 1E,F, Tables 1, 2). Neither were any of the other gross placental parameters significantly different. However, irrespective of gender, birth weight and placental weight were significantly lower in IUGR placentas by 34% (*p* < 0.001) and 33% (*p* < 0.001), respectively, than placentas of clinically normal pregnancies (Fig. 1E,F, Tables 1, 2). Statistically significant effects of disease were also found for the parameters surface area (20% smaller in IUGR) and the shortest diameter of the placental disc (11% shorter in IUGR, Table 1A,B).

**Table 1:** Descriptive Statistics of Macroscopic Parameters.

Descriptive Statistics				
Parameters	Group	Clinically normal	IUGR	Sex
		Mean ± SD (n)	Mean ± SD (n)	Mean ± SD (n)
GA (week)	♀	39.3 ± 1.0 (23)	37.8 ± 2.6 (22)	*38.6* ± *2.1 (45*
	♂	39.2 ± 0.7 (19)	37.2 ± 2.9 (18)	*38.2* ± *2.3 (37)*
		*39.3* ± *1.0 (42)*	*37.5* ± *2.7 (40)*	
BW (g)	♀	3361 ± 576 (23)	2314 ± 461 (22)	2849 ± *732 (45)*
	♂	3571 ± 453 (19)	2223 ± 551 (18)	2915 ± 833 (37)
		3456 ± *522 (42)*	2273 ± *493 (40)*	
PW (g)	♀	521 ± 124 (23)	341 ± 53 (22)	433 ± *130 (45)*
	♂	528 ± 106 (19)	362 ± 114 (18)	447 ± *136 (37)*
		524 ± *115 (42)*	350 ± *85 (40)*	
PW/BW	♀	0.153 ± 0.025	0.155 ± 0.042	*0.154* ± *0.033*
	♂	0.147 ± 0.019	0.167 ± 0.057	*0.157* ± *0.042*
		*0.150* ± *0.022*	*0.161* ± *0.048*	
SA (cm²)	♀	1149 ± 236 (23)	923 ± 211 (22)	1039 ± *247 (45)*
	♂	1143 ± 244 (19)	906 ± 287 (18)	1028 ± *284 (37)*
		1147 ± *234 (42)*	916 ± *241 (40)*	
ROUND	♀	1.18 ± 0.16 (23)	1.20 ± 0.12 (22)	*1.19* ± *0.14 (45)*
	♂	1.21 ± 0.14 (19)	1.22 ± 0.18 (18)	*1.21* ± *0.16 (*3*7)*
		*1.19* ± *0.15 (42)*	*1.21* ± *0.15 (40)*	
THICK (cm)	♀	1.7 ± 0.4 (23)	1.5 ± 0.3 (22)	*1.6* ± *0.4 (45)*
	♂	1.8 ± 0.4 (19)	1.5 ± 0.4 (18)	*1.7* ± *0.4 (*3*7)*
		*1.8* ± *0.4 (42)*	*1.5* ± *0.3 (40)*	
LD (cm)	♀	20.7 ± 2.9 (23)	18.7 ± 2.6 (22)	*19.7* ± *2.9 (45)*
	♂	20.8 ± 2.9 (19)	18.4 ± 2.6 (18)	*19.6* ± *3.0 (37)*
		*20.8* ± *2.8 (42)*	*18.5* ± *2.5 (40)*	
SD (cm)	♀	17.6 ± 1.9 (23)	15.6 ± 1.8 (22)	*16.6* ± *2.1 (45)*
	♂	17.3 ± 1.7 (19)	15.4 ± 3.0 (18)	*16.4* ± *2.6 (37)*
		*17.5* ± *1.8 (42)*	*15.5* ± *2.4 (40)*	

The table shows mean values (Mean), standard deviations (SD) and case numbers (n) of gestational age (GA), birth weight (BW) and the gross placental parameters placental weight (PW), ratio of PW to BW (PW/BW), surface area of the placental disc (SA), roundness of the placental disc (ROUND), thickness of the placental disc (THICK), longest diameter of the placental disc (LD), and shortest diameter of the placental disc (SD). PW/BW and Roundness are dimensionless factors; units of the other parameters are shown in brackets. The data are grouped into clinically normal placentas and placentas from pregnancies with intrauterine growth retardation (IUGR), and by gender (female ♀, male ♂). Aggregated data are in italics in the column Sex (aggregated by fetal gender) and the rows Group (aggregated by group).

**Table 2:** Statistical Testing (two-way ANOVA): The table shows the outcome of statistical testing (two-way ANOVA) for gestational age (GA), birth weight (BW) and the gross placental parameters placental weight (PW), ratio of PW to BW (PW/BW), surface area of the placental disc (SA), roundness of the placental disc (ROUND), thickness of the placental disc (THICK), longest diameter of the placental disc (LD), and shortest diameter of the placental disc (SD).

Testing	Clinically normal	IUGR	Female (♀)	Male (♂)
Comparison	♀/♂	♀/♂	clinically normal/ IUGR	clinically normal/ IUGR
Parameters				
GA (week)	ns	ns	ns	p < 0.05
BW (g)	ns	ns	p < 0.001	p < 0.001
PW (g)	ns	ns	p < 0.001	p < 0.001
PW/BW	ns	ns	ns	ns
SA (cm^2^)	ns	ns	p < 0.05	p < 0.05
ROUND	ns	ns	ns	ns
THICK (cm)	ns	ns	ns	ns
LD (cm)	ns	ns	ns	p < 0.05
SD (cm)	ns	ns	p < 0.05	p < 0.05

PW/BW and Roundness are dimensionless factors; units of the other parameters are shown in parentheses. Statistically significant outcome is indicated by p-values. Lack of statistical significance is indicated as not significant (ns). The first row groups the outcome by clinical groups (clinically normal, with intrauterine growth retardation (IUGR)) and then by gender (female (♀); male (♂)). The second row indicates for each column which variables were compared (comparison indicated by slash (/)).

The density of cell nuclei of villous trophoblast was determined by two measures, namely Mean Density (MD) and Nearest Neighbor Distance (NND). The MD was calculated by dividing the number of a given type of nuclei (PCNA-positive/PCNA-negative) by the surface area of the given branch (terminal/preterminal). Figure 1C,D shows Tukey plots and confidence intervals and Table 3 lists group-wise mean values of MD for both types of nuclei and branches. In the clinically normal group, the MD of PCNA-negative nuclei was found to be lower in males than females by 18.2% for terminal branches (bT0) and by 16.4% for preterminal branches (bT1). This effect was not observed in the IUGR group. However, irrespective of gender, a statistically significant increase in the MD of PCNA-negative nuclei was observed in the IUGR group as compared to the clinically normal group (Tables 3, 4). On the other hand, no statistically significant differences were observed in the MD of PCNA-positive nuclei.

**Table 3:** Descriptive Statistics. The table shows data of two different measures of spatial arrangement of nuclei of villous trophoblast, namely Mean Density of villous trophoblast nuclei (calculated by division of the total number of nuclei per branch through the surface area of the same branch) and Mean Nearest Neighbor Distance of villous trophoblast nuclei (determined for each individual nucleus).

TOPO	PCNA	Group	Clinically normal	IUGR	Sex
bT0/ bT1	Reactivity		Mean ± SD	Mean ± SD	Mean ± SD
*Mean Density of villous trophoblast nuclei (10* ^*−3*^ */µm²)*					
bT0	PCNA-neg.	♀	10.57 ± 2.37	13.78 ± 4.40	*12.14* ± *3.83*
	PCNA-neg.	♂	8.65 ± 2.06	13.77 ± 2.94	*11.14* ± *3.60*
			*9.70* ± *2.4*	*13.78* ± *3.76*	
bT1	PCNA-neg.	♀	9.19 ± 1.86	12.21 ± 3.84	*10.67* ± *3.33*
	PCNA-neg.	♂	7.68 ± 1.92	12.27 ± 2.49	*9.92* ± *3.19*
			*8.51* ± *2.01*	*12.24* ± *3.26*	
bT0	PCNA-pos.	♀	0.85 ± 0.66	1.43 ± 2.64	*1.13* ± *1.91*
	PCNA-pos.	♂	1.21 ± 0.93	0.71 ± 0.64	*0.96* ± *0.83*
			*1.01* ± *0.80*	*1.10* ± *2.02*	
bT1	PCNA-pos.	♀	0.64 ± 0.47	0.68 ± 0.77	*0.66* ± *0.63*
	PCNA-pos.	♂	0.82 ± 0.54	0.65 ± 0.56	*0.74* ± *0.54*
			0.72 ± 0.50	0.67 ± 0.67	
*Mean Nearest Neighbor Distance of villous trophoblast nuclei (µm)*					
bT0	PCNA-neg.	♀	4.06 ± 1.43	3.88 ± 1.44	*3.97* ± *1.44*
	PCNA-neg.	♂	4.37 ± 1.36	3.87 ± 1.43	*4.11* ± *1.45*
			*4.21* ± *1.45*	*3.87* ± *1.43*	
bT1	PCNA-neg.	♀	4.10 ± 1.43	3.92 ± 1.45	*4.01* ± *1.44*
	PCNA-neg.	♂	4.46 ± 1.46	3.99 ± 1.43	*4.22* ± *1.45*
			*4.28* ± *1.45*	*3.96* ± *1.44*	
bT0	PCNA-pos.	♀	8.56 ± 2.02	9.12 ± 2.17	*8.83* ± *2.09*
	PCNA-pos.	♂	7.81 ± 1.97	9.37 ± 2.02	*8.56* ± *2.01*
			*8.18* ± *1.99*	*9.24* ± *2.09*	
bT1	PCNA-pos.	♀	8.26 ± 1.96	9.95 ± 2.26	*9.06* ± *2.12*
	PCNA-pos.	♂	7.93 ± 1.88	9.64 ± 1.98	*9.64* ± *1.98*
			*8.09* ± *1.92*	*9.79* ± *2.12*	

The data are stratified by topological position of branches of the villous trees (TOPO, with terminal (bT0) and preterminal (bT1) position of the respective branch(es)) and by reactivity of the nuclei for proliferating cell nuclear antigen (PCNA, reactivity indicated by PCNA-positive (PCNA-pos) or PCNA-negative (PCNA-neg)). The data are shown as mean and standard deviation (SD) grouped by columns into clinically normal placentas and placentas from pregnancies with intrauterine growth retardation (IUGR), and by gender (female ♀, male ♂). Aggregated data are in italics in the column Sex (aggregated by fetal gender) and the rows Group (aggregated by group).

**Table 4:** Statistical Testing (two-way ANOVA). The table shows the outcome of statistical testing (two-way ANOVA) for the parameters listed in Table 3, namely Mean Density of villous trophoblast nuclei (calculated by division of the total number of nuclei per branch through the surface area of the same branch) and Mean Nearest Neighbor Distance of villous trophoblast nuclei (determined for each individual nucleus).

TOPO	PCNA	Clinically normal	IUGR	Female (♀)	Male (♂)
bT0/bT1	reactivity	♀/♂	♀/♂	clinically normal/IUGR	clinically normal/IUGR
*Mean Density of villous trophoblast nuclei (10* ^*−3*^ */µm²)*					
bT0	PCNA-neg.	p < 0.05	ns	p < 0.01	p < 0.001
bT1	PCNA-neg.	p < 0.05	ns	p < 0.01	p < 0.001
bT0	PCNA-pos.	ns	ns	ns	ns
bT1	PCNA-pos.	ns	ns	ns	ns
*Mean Nearest Neighbor Distance of villous trophoblast nuclei (µm)*					
bT0	PCNA-neg.	p < 0.001	ns	p < 0.001	p < 0.001
bT1	PCNA-neg.	p < 0.001	ns	p < 0.001	p < 0.001
bT0	PCNA-pos.	ns	ns	ns	ns
bT1	PCNA-pos.	ns	ns	p < 0.05	p < 0.05

The data are stratified in the first two columns by topological position of branches of the villous trees (TOPO, with terminal (bT0) and preterminal (bT1) position of the respective branch(es)) and by reactivity of the nuclei for proliferating cell nuclear antigen (PCNA, reactivity indicated by PCNA-positive (PCNA-pos) or PCNA-negative (PCNA-neg)). The outcome is also stratified by clinical groups (clinically normal, with intrauterine growth retardation (IUGR)) and then by gender (female ♀, male ♂). The second row indicates for each column of outcomes which variables were compared (comparison indicated by slash (/)). Statistically significant outcome is indicated by p-values. Lack of statistical significance is indicated as not significant (ns).

The second measure of the density of cell nuclei of the villous trophoblast was the NND. It was measured as Euclidean distance from each nucleus to its closest neighboring nucleus. Per placenta 6012 (bT0) and 2736 (bT1) PCNA-negative nuclei and 180 (bT0) and 252 (bT1) PCNA-positive nuclei were analyzed. Figure 1A,B shows Tukey plots and confidence intervals and Table 3 lists group-wise mean values of NND for both types of nuclei and branches. The NND of PCNA-negative nuclei was found to be shorter in females than males by 7.1% for terminal branches (bT0) and by 8.1% for preterminal branches (bT1) in the clinically normal group. This effect was not observed in the IUGR group. However, irrespective of gender, a statistically significant decrease in the NND of PCNA-negative nuclei on both branches bT0 and bT1 was observed in the IUGR group as compared to the clinically normal group (Tables 3, 4). On the other hand, no gender specific differences were observed in the NND of PCNA-positive nuclei, neither in the clinically normal group nor in the IUGR group (Tables 3, 4). Only the NND of PCNA-positive nuclei on bT1 branch was significantly longer in the IUGR group than the clinically normal group, irrespective of gender.

Table 5 summarizes important parameters of the statistical tests of the present study with regard to gender specific differences for the measures birth weight (BW), placenta weight (PW), MD and NND in the clinically normal group. The presented parameters for MD and NND relate to PCNA-negative nuclei at bT0 branch.

**Table 5:** The table shows the estimates of effect size (Cohen’s d), the sample size and the observed statistical power for the parameters birth weight (BW), placenta weight (PW), mean surface density of PCNA-negative trophoblast nuclei (MD) and nearest neighbor distance of PCNA-negative nuclei (NND).

Parameters	Cohens *d*	Sample Size	Power	♀/♂
BW (g)	0.4	42	0.24	ns
PW (g)	0.1	42	0.06	ns
MD	0.8	42	0.77	p < 0.05
NND	0.2	12024	$0.\bar{9}$	p < 0.001

Only the data of the control group were considered for calculating these estimates. The last column indicates whether a significant difference was observed between male and female.

## Discussion

The present study establishes the density of PCNA-negative cell nuclei of term human villous trophoblast as a physiologically sexually dimorphic microscopic feature of human placentas. Given that the higher weight of male than female placentas as epidemiological hallmark of sexual dimorphism is a general feature of normal human placentation and is occurring independent of maternal age, race and other demographic parameters^17^, the findings of the present study likely are representative.

**Figure 2:**
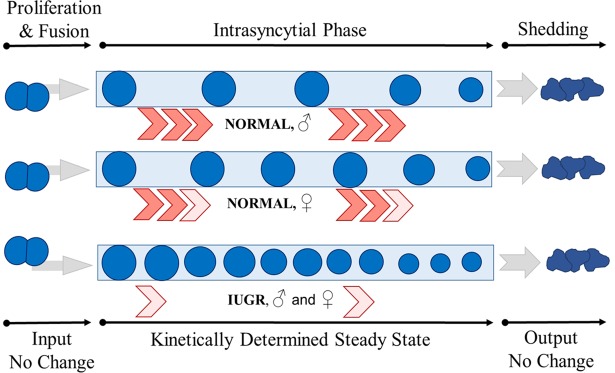
The figure illustrates the principal idea of the concept of post-fusion control of syncytial structure and function proposed in the present study. The left side starts with input of cellular elements (blue dots symbolize nuclei, but all organelles are imported likewise) by cyto-syncytial fusion based on constant proliferation. The right side illustrates output of aged nuclei and organelles by shedding into the maternal circulation. The three pale blue rectangles symbolize syncytiotrophoblast and the effect of rate of progression (symbolized by red angles; number and color intensity symbolize the rate of progression) through the intrasyncytial phase on the density of organelles (nuclei shown are exemplary of all intrasyncytial organelles). The size of nuclei inside syncytiotrophoblast symbolizes ageing with smaller dots for more senescent nuclei. The proposed concept develops on actual concepts^37,38^ on turnover and steady state of human villous trophoblast. It adds the aspect of time spent on passage of cytotrophoblast material (here: nuclei) through the syncytium and thereby interprets apparent changes of nuclear density as a consequence of variability of passage time (kinetic determination of apparent features of steady state).

Since only the PCNA-negative (i.e. non-proliferative) cell nuclei of villous trophoblast were subject to this effect, the unique bi-layered architecture of villous trophoblast as an epithelium comes into focus. It is clear that most of the PCNA-negative cell nuclei are part of the apical trophoblast layer, the syncytiotrophoblast^35,37^. Syncytiotrophoblast is the central tissue organizing materno-fetal transport and traffic^30^ and the present study shows that this is the sexually dimorphic part of villous trophoblast. Syncytiotrophoblast is a huge polarized cellular mass maintained during pregnancy by post-proliferative cyto-syncytial fusion of cytotrophoblast units into the syncytiotrophoblast^30,37,38^. Aged nuclei and organelles of syncytiotrophoblast^39^ are finally shedded into the maternal circulation^37,38^. Syncytiotrophoblast is thus a kind of steady state tissue with intrasyncytial components (cell nuclei, organelles, vesicles, membranes) existing in between cyto-syncytial fusion and syncytial shedding. Since the density of PCNA-positive cell nuclei (most of them prior to cyto-syncytial fusion^35,37,38^) was not sex-related, the sexual dimorphism of density of PCNA-negative nuclei is not just an outflow of sexually dimorphic trophoblast proliferation. An intriguing concept integrating the actual views on syncytiotrophoblast^37,38^ and the data of the present study is a post-fusion kinetic control of syncytial structure by variation of the transsyncytial passage time of cytotrophoblast material (see Fig. 2). This can possibly be understood as a kind of brake system controlling the kinetics of intrasyncytial passage of PCNA-negative cell nuclei. It could be active at its latest at the point of shedding and/or at any intrasyncytial passage point prior to it. In such a concept, one or more of the (many and still very incompletely understood^37,38^) sequential steps during ageing of syncytial organelles^39^ towards shedding would function as regulated rate limiting step(s). Through such rate limiting steps, syncytial passage time of cell nuclei could be elongated while proliferation, cyto-syncytial fusion and shedding could stay fairly constant (see Fig. 2).

According to current views^37,38,40–42^, syncytiotrophoblast consists of post-fusion cytotrophoblast nuclei/organelles which are progressively passivated by ageing^39^ including many features of apoptosis^40–42^, and substantial reduction of transcription^43^ after syncytial fusion. In the frame of these concepts, regulatory influences are predominantly expected to be proliferation-driven, while the elements included in syncytiotrophoblast progress towards toward shedding without control like water in a waterfall. The concept of trophoblast proliferation as the only regulatory driver of the passively ageing syncytium^41,42^ is challenged by the physiological sexual dimorphism and the findings in IUGR placentas described in the present study. In the absence of sexually dimorphic proliferation, the sexually dimorphic syncytial steady state found in the present study is best explained by a type of post-fusion regulation instead of pre-fusion proliferative control of inflow into syncytiotrophoblast. It is obvious that more pronounced control of syncytial passage time would also explain the state seen in IUGR placentas of the present study, again without a corresponding biologically adequate change in the proliferative compartment of villous trophoblast (see Fig. 2).

Regulation of syncytial function during the post-proliferative phase of villous trophoblast is a new paradigm we propose based on the data of the present study (see Fig. 2). Its existence can have far reaching consequences in various areas of placental research.

### Oxidative stress, reactive oxygen species and villous trophoblast

Occurrence of markers of oxidative stress and also antioxidant levels become increasingly important in placental research^44^ and are sexually dimorphic^45^. This was shown in placental homogenates and thus not tissue-specific for villous trophoblast. Nevertheless, villous trophoblast is rich in organelles and a mass tissue of the placenta. Villous trophoblast delivers a major part of the contents of placental homogenates. Antioxidant capacity was higher in male than in female placentas while detection of adducts due to mitochondrial production of reactive oxygen species behaved reciprocally^45^. Furthermore, these studies propose that higher male than female loss of anti-oxidant capacity in case of adversity (here: maternal obesity) could be a mechanism by which the higher clinical risk in male pregnancies is mediated^45^. Similar to the data of the present study on IUGR, sexual dimorphism becomes diminished or even extinguished in case of adversity^45^. Increased levels of oxidative stress were found in many pregnancies with a complicated clinical course^46–49^, though usually without analysis of sexual dimorphism. The concept of kinetic post-fusion regulation of syncytiotrophoblast proposed above would imply that the higher density of cell nuclei in female villous trophoblast coincides with a more mature/senescent stage of many organelles and nuclei in female trophoblast than in male trophoblast. Higher levels of adducts of reactive oxygen species could thus not only reflect higher levels of reactive oxygen species but could also occur with normal levels of reactive oxygen species due to longer duration of exposure during a longer intrasyncytial passage time in the presence of decreasing (due to progressive ageing) antioxidant levels.

### Sexually Dimorphic mRNA Levels in Syncytiotrophoblast

There are reports on sexually divergent gene expression in the human placenta^50–52^, also on gene expression explicitly related to villous trophoblast^33^. However, a sexually dimorphic regulation of gene expression is difficult to imagine in a constantly ageing tissue like syncytiotrophoblast with low or even not higher than residual transcription levels. The residual transcription levels in syncytiotrophoblast required an extremely sensitive and sophisticated approach for their detection^43^. After cyto-syncytial fusion, the transcription level rapidly decreases and syncytiotrophoblast is more and more dependent on its existing mRNA content. It is questionable whether the cellular mechanisms of genetic expression control are still fully active in this “para-apoptotic”, ageing multinuclear cellular construct. Sexually dimorphic levels of mRNA could thus (i) be inherited by preexisting differences in cytotrophoblast through cyto-syncytial fusion, or (ii) they might be caused by elongated passage time and differential decay rates of the various mRNAs in the syncytiotrophoblast after cyto-syncytial fusion. Thus, such differences of specific mRNA levels potentially could not only be due to differential gene expression but also to differential decay rates of mRNAs inside syncytiotrophoblast.

### Sexual Dimorphism in Animal Models

There are recent data of animal models, which shed light on the importance of trophoblast for sexual dimorphism and on the placental response to prenatal insults^53^ and stressors like hypoxia^54^. While the considerable species divergences in placentation make it difficult to match molecular and morphological data one on one to human placentation, the common denominator is the importance of trophoblast for sexual dimorphism. Potentially, sexual dimorphism of trophoblast is a general feature of reproductive biology and not limited to human placentation. This also supports the view that trophoblast – potentially also in human placentation – could mediate sexually divergent degrees of resilience^53^ to the stressors provided by pregnancy complications, especially IUGR.

So far, basic research on IUGR is focusing on the first trimester and generally on early placentation. The role of trophoblast specified in the present study and supported by animal models now also justifies a more diligent view on trophoblast in late pregnancy, where disease is becoming clinically symptomatic. This time window late in pregnancy could harbor so far undiscovered options for pro-resilient therapeutic interventions having trophoblast as target tissue.

### The Syncytiotrophoblast in Placental (Histo-)Pathology

The substantially higher density of nuclei in syncytiotrophoblast of IUGR cases documented by the present study could potentially be one of the factors leading to the so-called Tenney Parker changes (syncytial knots) in placental histopathology^30^. These changes consist of apparent syncytial knots and bridges with many syncytial nuclei which become visible in 2D sections. However, the majority of this pattern is considered a 2D sectioning artifact related to varying section thickness, branching of the villous surface and nuclei content of syncytiotrophoblast^55,56^. Tenney-Parker changes occur in the histopathology of various obstetrical complications, especially in IUGR and Preeclampsia^30^. While the present study was not designed to prove that the increased density of nuclei in syncytiotrophoblast is the cause of this phenomenon, it is nevertheless likely that the increased density of nuclei in syncytiotrophoblast (in 3D) is contributing to the occurrence of syncytial knots in 2D sections. Since Tenney-Parker changes are a widespread observation in many obstetrical syndromes, it is possible that the increase of the density of nuclei in villous trophoblast described for IUGR in the present study could be equally widespread, and is reflective of a general pattern of reaction of villous trophoblast to stress in pregnancy.

### The Syncytiotrophoblast in Epidemiology and Prenatal Programming

So far, syncytiotrophoblast did not play a major role in studies on prenatal programming. Sexual dimorphism of syncytiotrophoblast has been detected in the present study at exceptionally high statistical power (Table 5). Actually, there is no other macroscopic or microscopic parameter available, which can detect the small effects of sexual dimorphism on the placenta with comparable power.

## Material and Methods

### Clinical Groups and Placental Tissue

The present study used 42 placentas of clinically normal pregnancies and 40 placentas of pregnancies with intrauterine growth restriction (IUGR). Peripheral villous trees were analyzed by 3D microscopy. All placentas were collected at the Department of Obstetrics and Gynecology of the hospital “Dritter Orden”, Munich, Germany. Core clinical data and gross anatomical placental data are shown in Table 1.

The ethics board of Ludwig-Maximilians-University of Munich (LMU Munich), under the numbers 084–11 and 478–12, approved all investigations. All methods and procedures were performed in accordance with relevant guidelines and regulations.

Placentas were collected after informed consent of the mothers/parents was obtained. Intrauterine growth restriction was clinically diagnosed if the growth parameters of the fetus determined by ultrasound (e.g. femur length, abdomen and head circumference) were above the 10th growth percentile during the first two trimesters and then dropped below the 10th growth percentile. The obstetricians made this diagnosis during routine clinical monitoring. The morphological examiners were aware of the gestational age, fetal weight and whether there was a clinical diagnosis of IUGR, but were blinded to any other clinical data. The ethical vote constrained use of clinical data to data relevant for group allocation with few exceptions like birth weight and gestational age. Placentas of patients with a combination of IUGR and symptoms of preeclampsia were excluded in this study.

### Tissue Sampling and Tissue Processing

All placentas were cooled at 4 ^◦^C immediately after birth and transported under constant refrigeration to the Department of Anatomy II of LMU Munich. Sampling of tissue was per- formed using an established systematic-and-random procedure^35,36^.

A point pattern was projected onto the chorionic surface of the placenta. The first sampling site was determined as being the first point falling fully inside the placental disc. By appropriate spacing (every third or fourth point, depending on the size of the placenta) it was possible to determine six sampling sites on each placental disc. Each sampling position was labelled with a small numbered pin and the chorionic surface with all pins in place was photographed with a ruler included in the picture.

Sampling of isolated peripheral villous trees for analysis with 3D microscopy was performed at the midpoint between sampling sites 3 and 4. At this position a piece of tissue with an edge length of about 2 to 3 cm and encompassing the entire thickness of placenta was taken and rinsed in physiological saline at 4 ^◦^C. Free bushes of peripheral villi (2–4 mm length) were identified under a binocular microscope and carefully isolated. Care was taken to touch the small bushes only at larger stem villi and to keep the peripheral parts of the bushes freely floating. The excised small bushes were then transferred to 4.5% formaldehyde (Roti-Histofix, Carl Roth, city, state, country) and immersion fixed for at least 24 h while still freely floating in fixative. It is of note that all steps of fixation, dehydration, staining, immunohistochemistry and embedding using these small bushes were done with the bushes freely floating in the incubation solutions or embedding mixtures. Maximum care was taken to avoid deformation of villous trees at any point of the procedure.

### Immunohistochemistry

The isolated peripheral villous trees were washed in tap water for 60 min, then briefly in distilled water, transferred through an ethanol step gradient (50%, 70%, 80%, 96%, 5 min in each step of the gradient) into 100% ethanol and then transferred back from 100% ethanol to distilled water through the same gradient. This passage to ethanol and back to water was undertaken to open membranes of the fixed tissue for better antibody penetration (implemented as a result of empirical optimization of tissue penetration). Then the villous trees were shortly washed in cold 1 N HCl. Antigen retrieval was supported by incubation for 10 min in 1 N HCl at 60 ^◦^C, followed by washing in distilled water (3 × 10 min) and PBS at room temperature overnight. Endogenous peroxidases were blocked (3% H_2_O_2_ for 20 min), followed by washing with PBS two times (3rd time PBS with 0.1% Tween). The villous trees were incubated with primary antibody (mouse anti-PCNA, 1:9000 in PBS buffer; article no. 180110; Invitrogen) for 60 min at room temperature, then overnight at 4^ ◦^C and on the next day for another 60 min at room temperature, then washed for 90 min in PBS. Blocking solution, solvent for 2nd antibody and the streptavidin complex were obtained from the ZytoChem-Plus HRP kit (article No.HRP125; Zytomed Systems, Berlin, Germany) and used according to the kit protocols. Samples were incubated in the solution containing the secondary antibody for 15 min, then washed for 3 × 15 min in PBS, incubated with streptavidin complex for 15 min and again washed for 3 × 15 min in PBS. Visualization was achieved by peroxidase detection using diaminobenzidine (15 min, DAB) as substrate and then washed in distilled water (3 × 15 min). Counterstaining with diluted hematoxylin (1:8 in distilled water) was achieved by incubation in the stain solution (7 min), followed by bluing in tap water for 10 min and washing in distilled water again. Preparations were passed through an increasing alcohol step gradient to xylene and mounted on concave slides in DPX (article No. 360294 H; Prolab VWR, Darmstadt, Germany) under cover slips. Care was taken to infiltrate the isolated villous trees slowly and completely with DPX prior to mounting. This was achieved by infiltrating the isolated villous trees with a mixture of xylene/DPX 1:1 (v/v) for 4 h prior to transfer and mounting on the concave slides in DPX. Once mounted on the concave slides, the preparations had a true 3D character and were free from compressive contact with the concave slide or cover glass (Supplementary Figs 1, 2).

### Topological Microscopy of Isolated Peripheral Villous Trees

All peripheral villous trees were traced by mouse clicking and mouse wheel operations with Neurolucida software (version 11.02; MBF Bioscience, Williston, VT, USA) under a brightfield microscope using a 20x objective with the working direction from the proximal toward the terminal end of the peripheral villous tree using established procedures^35,36^. The diameter of branches was continuously determined as a frustum around the center point. The focus plane showing the largest diameter of a branch segment was identified as the best representative of the center point in 3D^35,36^. All center points placed in 3D were automatically connected to a continuous center line by the software, providing an accurate 3D skeleton for topological tree analysis (Supplementary Fig. 2). Tree ordering (branch topology) was set to “Terminal Distance Ordering” in the Neurolucida software, because the terminal end was the biologically defined end of the isolated peripheral villous trees. This setting classified branches according to their distance in nodes from the terminal end of the villous tree. The parts of villi connecting two nodes or connecting a terminal end with a node were named branches (b). Branches were further classified by their distance to the nearest terminal end (bT, with the T indicating classification by terminal distance). The distance was measured by the number of nodes to the nearest terminal end, i.e., bT0 (terminal end), bT1 (one node apart of the next terminal end) and bT2 (two nodes apart of the next terminal end), see also^35,36^.

By continuous focusing through the villous trees the roundish, large epithelial nuclei of villous trophoblast became visible on the branch surface (Supplementary Fig. 2) and could be unequivocally discriminated from each other. Furthermore, they could easily be distinguished from smaller and more elongated stromal or endothelial nuclei. All PCNA-positive and PCNA-negative nuclei were separately marked by the software by clicking on the midpoint of each nucleus in best focus with the mouse. Each nucleus was sampled once and as a whole (in contrast to thin sections where only nuclear profiles, but not whole nuclei are analyzed). The software automatically determined the three dimensional (XYZ) coordinates of each midpoint of a nucleus. This data was later used for determination of the branch-specific density of villous trophoblast nuclei at the villous surface. Furthermore, the software automatically associated each nucleus with its branch of the villous tree by allocation to the nearest centerline of the tree.

This process generated a digital 3D replica of the peripheral villous tree under investigation concurrently with the process of tracing and identification of nuclei (Supplementary Fig. 2). By detection of PCNA, the nuclei could nevertheless be generally associated with syncytiotrophoblast (most are PCNA-negative) and cytotrophoblast (PCNA-positive)^35^. For the sake of our analysis cyto- and syncytiotrophoblast are treated and conceptualized as a single epithelial compartment (villous trophoblast).

Investigations were performed using two microscope systems with 20x objectives for evaluation. The first one consisted of an Axioskop microscope (Zeiss, Goettingen, Germany) with a motorized XYZ specimen stage (Maerzhaeuser, Wetzlar, Germany), LEP MAC6000 XYZ 3-axis stage controller (Ludl Elec- tronic Products, Hawthorne, NY, USA), focus encoder (Type MT 1271; Heidenhain, Traunreuth, Germany) and color digital camera (3/40 CCD chip 1,92 MP, 1600 1200 pixel; MBF Bioscience). The second consisted of a BX50 microscope (Olympus, Tokyo, Japan) with motorized XYZ specimen stage (MBF Bioscience), LEP MAC6000 XYZ 3-axis stage controller (Ludl Electronic Products), focus encoder (Type MT 1271; Heidenhain) and color digital camera (1/20 CCD chip, 1392 × 1040 pixel, MBF Bioscience).

Data were analyzed using the software Neurolucida Explorer (version 11.09; MBF Bio- science) using the options “branching structure analysis” and “marker analysis”. Nearest neighbor distances of villous trophoblast nuclei were automatically determined by the soft- ware for each nucleus which had been labeled during the microscopic analysis. Densities of villous trophoblast nuclei were calculated by dividing the number of nuclei per branch by the surface area of the branch. This was separately done for nuclei which were immune-positive for PCNA (PCNA-positive) and for nuclei which were immune-negative for PCNA (PCNA-negative).

### Statistical Analysis

Following clinical and gross anatomical parameters were included in analysis: gestational age (GA), birth weight (BW), placenta weight (PW), the ratio PW/BW, surface area (SA), roundness of the placental disc (Round), thickness of placenta (Thick), the shortest diameter (SD) and the longest diameter (LD). The data were grouped according to gender (female, male) and clinical outcome (normal, IUGR), resulting in four different clinical groups. Table 1 lists the group and gender means for these macroscopic parameters. A two-way analysis of variance (ANOVA) was performed on all the macroscopic parameters to investigate the interaction between the grouping variables. Pairwise comparison of means was done according to Tukey. Table 2 lists the results of the pairwise comparisons.

### Mean Density of villous trophoblast nuclei

For each placenta mean values for the density of villous trophoblast nuclei were calculated by dividing the number of nuclei identified on the surface of branches by the surface area of the respective branch. The mean density was calculated for both types of nuclei, PCNA-positive and PCNA-negative, and for each branch type, bT0 and bT1. The mean values together with standard deviations are given in Table 3.

The mean density data were transformed to logarithmic scale as the frequency histograms were skewed to right for all four groups. After transformation, the Shapiro-Wilk normality tests were non-significant for all the groups (data not shown). A two-way analysis of variance was performed on the transformed data with gender (female, male) and disease (normal, IUGR) as grouping variables. A significant interaction between the grouping variables was found only for the PCNA-negative nuclei. The results for bT0 and bT1 were F (1, 78) = 4:25; *p* = 0:042 and F (1, 78) = 4:30; *p* = 0:041, respectively. The subsequent pairwise comparisons of means according to are shown in Table 4.

### Mean Nearest Neighbor Distance of villous trophoblast nuclei

The nearest neighbor distance (NND) for a nucleus is the Euclidean distance to its closest neighboring nucleus^35^. It was derived for each nucleus from the 3D coordinates of all nuclei. Like mean density, mean NND was calculated for both type of nuclei and for each type of branch. The mean values together with standard deviations are given in Table 3.

The NND data had a right skewness in frequency distribution, too. Thus, it was transformed to logarithmic scale. Subsequent Shapiro-Wilk normality tests were still highly significant (data not shown). We believe that the large sample size for NND (*n* < 10000) creates a very high statistical power for the normality test. Hence, even subtle deviations from normal distribution result in significant normality test. It is known that ANOVA is robust to small departures from normal distribution. Also, heterogeneous variances among groups have little effect on the significance level of ANOVA, if the groups have equal sample sizes^57^. However, the significance level is seriously affected by a combination of non-normality and heterogeneous variances in unbalanced studies^57^. Therefore, a balanced study was designed for NND by randomly drawing equal number of samples for all four groups. In a separate study, the findings in this paper were validated by gradually and randomly decreasing the number of cases. They were found to be stable against random data reduction in balanced studies.

After performing two-way ANOVA on NND, a significant interaction was found between gender and disease for PCNA-negative nuclei. The results of interaction term for bT0 and bT1 were F (1, 24044) = 60:03; *p* < 0:001 and F (1, 10940) = 29:62; *p* < 0:001, respectively. The subsequent pairwise comparisons of means according to Tukey are shown in Table 4.

## Supplementary information

Supplementary Information
