# Supplementary material for: The Density of Cell Nuclei at the Materno-Fetal Exchange Barrier is Sexually Dimorphic in Normal Placentas, but not in IUGR
Source: Sci Rep. 2019 Feb 20;9:2359. doi: 10.1038/s41598-019-38739-9 (PMC6382753; doi:10.1038/s41598-019-38739-9)
Supplement: Supplementary file 1 — Supplementary Information [file 41598_2019_38739_MOESM1_ESM.pdf]

Supplementary Information

The Density of Cell Nuclei at the Materno-Fetal Exchange Barrier is Sexually Dimorphic in Normal Placentas, but not in IUGR

Nirav Barapatre[1a], Eva Haeussner[1a], David Grynspan[b], Christoph Schmitz[a], Franz Edler von Koch[2c], Hans-Georg Frank[2a*]

[a] LMU Munich, Faculty of Medicine, Institute of Anatomy, Chair of Neuroanatomy, Munich, Germany.

[b] University of Ottawa, Department of Pathology and Laboratory Medicine, Ottawa, Canada.

[c] Clinic for Obstetrics and Gynecology Dritter Orden, Munich, Germany.

1 Both authors contributed equally as first authors.

2 Both authors contributed equally as principal investigators.

* Corresponding author: Chair of Neuroanatomy, Institute of Anatomy, Faculty of Medicine, LMU Munich, Pettenkoferstr. 11, 80336 Munich, Germany, email: hans-georg.frank@med.uni-muenchen.de

---

Supplementary Figures

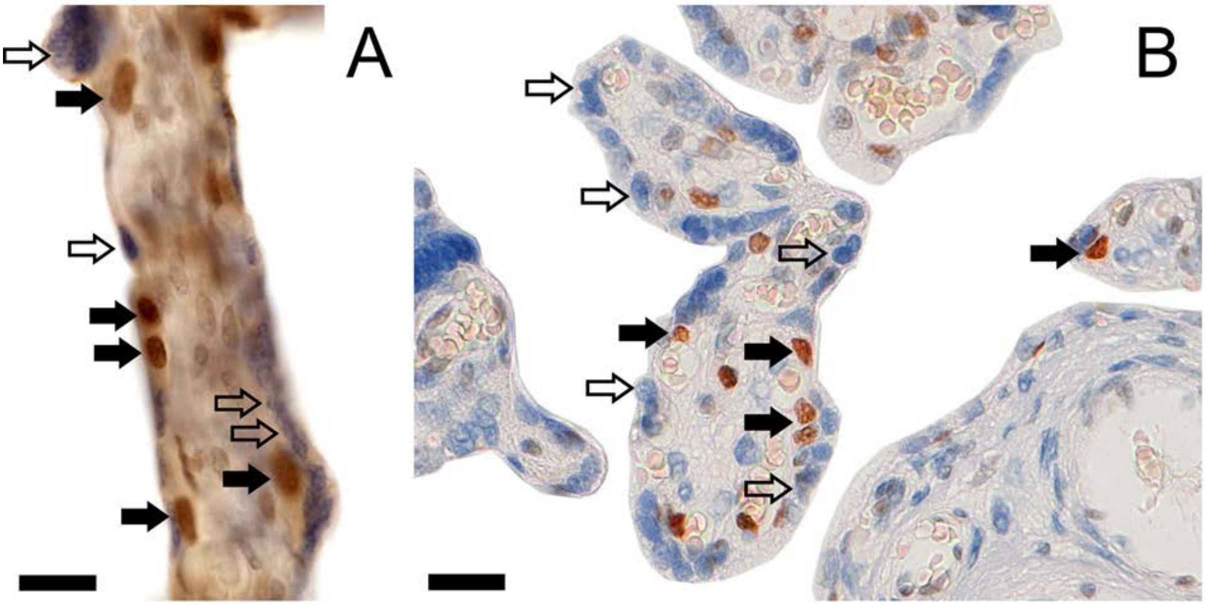

Supplementary Figure 1: Immunochemical detection of proliferation cell nuclear antigen (PCNA). (A) Exemplary microscopic view (one visual field and one focus level only) of a peripheral villous tree during 3D microscopy. PCNA-positive (brown nuclei; dark arrow) and PCNA-negative (blue nuclei; clear arrow) trophoblast nuclei can be recognized on the villous surface. Scale bar equates to 18µm. (B) Exemplary microscopic view of a thin 2D histological section of villous trees. PCNA-positive (brown nuclei; dark arrow) and PCNA-negative (blue nuclei; clear arrow) trophoblast nuclei can be recognized on the villous surface. Scale bar equates to 25µm. With permission, taken from [35].

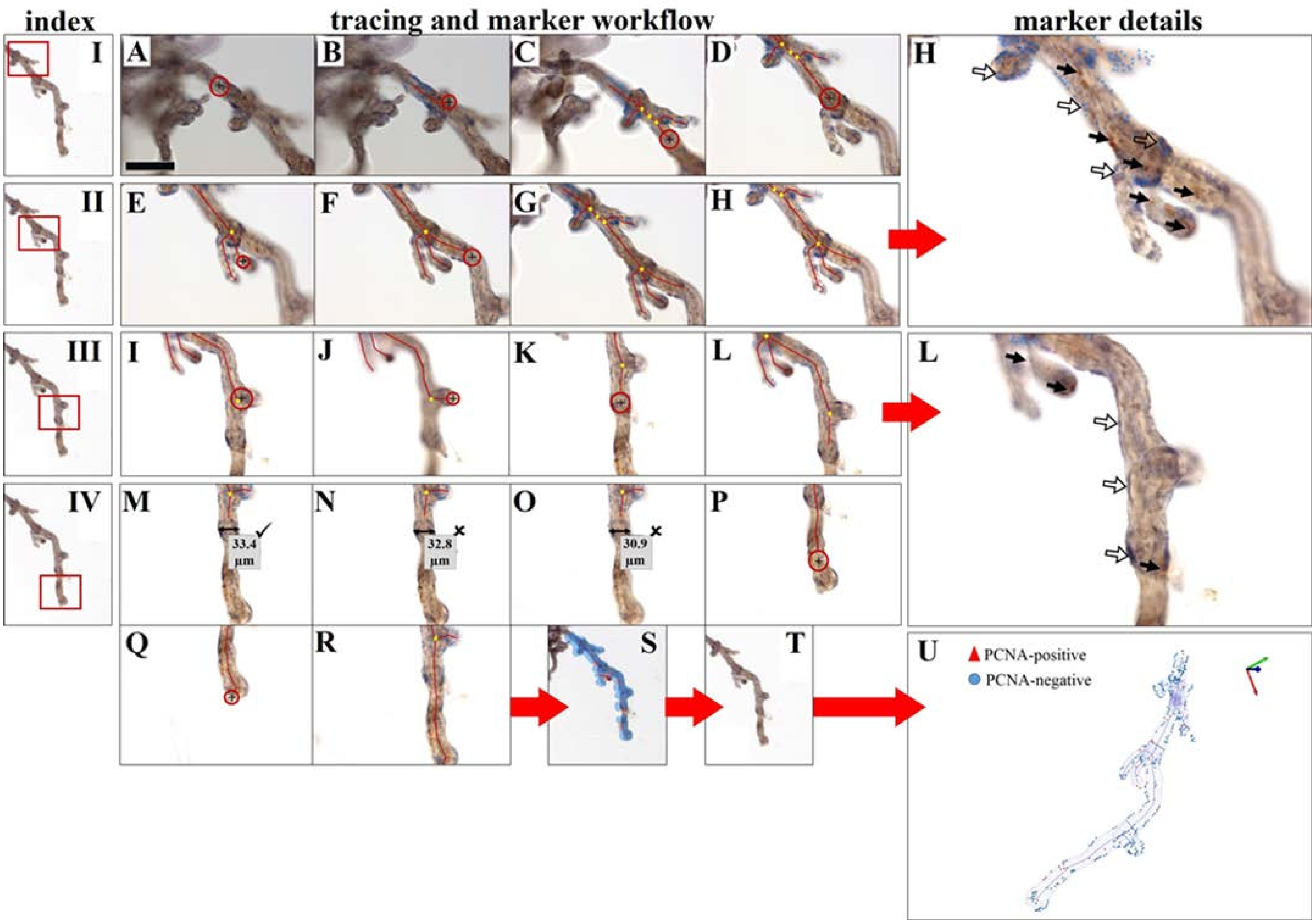
index
tracing and marker workflow
marker details
I
II
III
IV
A
B
C
D
E
F
G
H
I
J
K
L
M
N
O
P
Q
R
S
T
H
L
U
33.4 µm
32.8 µm
30.9 µm
PCNA-positive
PCNA-negative

Supplementary Figure 2: Illustration of the computer-assisted workflow to generate 3D replica of villous trees combined with trophoblast marker analysis. Zoom in for details as required. (I-IV) show a local index of the region were the actual measurements are localized. (I) corresponds to (A-D), (II) corresponds to (E-H), (III) corresponds to (I-L), and (IV) corresponds to (M-R). (A-R) show the tracing procedure (red lines and cycles; branching nodes are marked as yellow dots) with parallel labeling of nuclei markers on the villous surface. Focusing through the x/y/z-axis the villous tree is traced in 3D and in parallel the trophoblast nuclei become labeled by placing markers with mouse clicks on their respective x/y/z position. The tracing procedure starts by adapting the red cycle with the mouse wheel function to the diameter of the villous segment (A), thereby keeping an eye on finding the z-level with highest diameter (exemplary shown in M-O) of the villous while seeing the nuclei as sharp as possible at the sides of the villus. This ensures positioning of the centerline in the middle of the villus. The red circle is then moved a short distance (B) repetitively (B-R) while always adapting the diameter of the red circle to the diameter of the villus with the mouse wheel. By this, the tree is followed and traced. All visible trophoblast nuclei can be labeled by right-click as being PCNA-positive or PCNA-negative nuclei. For the purpose of clarity of the figure, only examples of labeling of nuclei on the surface of the villous tree of the figure are shown exemplary only. The image enlargements (H,L) show details in the original view provided during the 3D microscopic routine (PCNA-positive: dark arrow; PCNA-negative: clear arrow). (S) shows the region recorded by this topological 3D microscopic routine, (T) shows a total view of the tree without labeling, and (U) shows the replica which was produced during this examination by the Neurolucida software in the computer. The labeling of nuclei is not complete, but exemplary in this demonstration case. PCNA-negative nuclei are blue, PCNA-positive nuclei are red. The centerline shows the middle line of the villous tree and nodes, the wireframe shows the surface area of the tree. The scale bar in (A) is 25 µm and is valid for (A-R), and 250 µm for (I-IV; S,T). With permission, taken from [35].
